# Scalable and High-Throughput Top-Down Manufacturing of Optical Metasurfaces

**DOI:** 10.3390/s20154108

**Published:** 2020-07-23

**Authors:** Taejun Lee, Chihun Lee, Dong Kyo Oh, Trevon Badloe, Jong G. Ok, Junsuk Rho

**Affiliations:** 1 Department of Mechanical Engineering, Pohang University of Science and Technology (POSTECH), Pohang 37673, Korea; leetj@postech.ac.kr (T.L.); chihunlee@postech.ac.kr (C.L.); ehdry12390@seoultech.ac.kr (D.K.O.); trevon@postech.ac.kr (T.B.); 2 Department of Mechanical and Automotive Engineering, Seoul National University of Science and Technology, Seoul 01811, Korea; jgok@seoultech.ac.kr; 3 Department of Chemical Engineering, Pohang University of Science and Technology (POSTECH), Pohang 37673, Korea

**Keywords:** nanofabrication, metasurfaces, scalable, high-throughput, large-scale, metadevices, lithography, nanopatterning, top-down fabrication

## Abstract

Metasurfaces have shown promising potential to miniaturize existing bulk optical components thanks to their extraordinary optical properties and ultra-thin, small, and lightweight footprints. However, the absence of proper manufacturing methods has been one of the main obstacles preventing the practical application of metasurfaces and commercialization. Although a variety of fabrication techniques have been used to produce optical metasurfaces, there are still no universal scalable and high-throughput manufacturing methods that meet the criteria for large-scale metasurfaces for device/product-level applications. The fundamentals and recent progress of the large area and high-throughput manufacturing methods are discussed with practical device applications. We systematically classify various top-down scalable patterning techniques for optical metasurfaces: firstly, optical and printing methods are categorized and then their conventional and unconventional (emerging/new) techniques are discussed in detail, respectively. In the end of each section, we also introduce the recent developments of metasurfaces realized by the corresponding fabrication methods.

## 1. Introduction

Metasurfaces are planar arrays of subwavelength nanostructures. Thanks to their ultra-thin, small, and lightweight features, they are one of the most promising candidates for replacing bulk optical components and devices. Metasurfaces not only enable the miniaturization of existing bulk optical devices, but also show enhanced performance. Conventional optical components such as glass lenses basically employ the refraction of light, which limits their optical performance. Metasurfaces, on the other hand, have been considered as a promising candidate for the replacement of conventional lenses. They have been applied as high-resolution ultrathin lenses [1,2], as well as superlenses and hyperlenses that overcome diffraction limits [3,4,5]. Metasurfaces can also play the role of color filters, selectively transmitting or reflecting the specific wavelengths of incident light to produce the desired colors [6,7,8,9,10,11,12,13]. Using a tunable polarization-dependent structural coloration, cryptography has been demonstrated to hide multiple pieces of information in one metasurface [6,9]. In addition to color filters, holography is another coloration application of metasurfaces. In metaholograms, each meta-atom on the metasurface is designed to resonate the incident light at a certain visible frequency, so the desired image can be displayed [14,15,16,17,18,19,20]. K. Kim and G. Yoon have also shown that metaholograms can be realized at a large scale using nanoimprint lithography (NIL) [16]. Through the miniaturization and performance enhancement of bulk optical components, metasurfaces have been used in imaging applications such as resolution-enhanced fluorescence microscopy [21,22] and laser scanning microscopy [23]. Furthermore, the extraordinary optical properties of metasurfaces enable the perfect absorption of light [24,25,26,27,28,29,30], and light manipulating applications such as beam splitting [31,32,33], verification and enhancement of the spin Hall effect [34,35,36], asymmetric transmission [37], multifunctional waveguides [38], and other light-manipulation applications [39,40,41,42,43,44,45,46,47,48,49]. In addition, the researches on various materials of the metasurfaces have been actively reported: a metasurface-based solar reflector using vanadium dioxide (VO_2_) [43,50,51,52,53], a study on the manufacturing of graphene oxide film for anisotropic photoresponse [54], and the scalable nanostructures of molybdenum disulfide (MoS_2_) [55]. However, in order for existing bulk optical devices or components to be completely replaced with metasurfaces, those metasurfaces must be fabricated consistently with a large area and high throughput. In other words, mass-production technology should be developed further for commercialization and the application of metasurfaces into our daily lives. 

Focus needs to be on the manufacturing methods that allow the large scale and high-throughput fabrication of metasurfaces for mass-production. Although various nanofabrication techniques exist, ones that meet the demands of both large-area and high-throughput patterning simultaneously are limited. In this review, we classify the various methods into two parts: optical and printing methods. The optical methods are further divided into conventional photolithography and interference lithography, with each being discussed in more detail. Photolithography is the most commonly used optical method for metasurface manufacturing. It is done by transferring a light through a photomask onto a photo-sensitive material. The laser interference lithography (LIL) technique is a mask-less manufacturing method and uses the interference pattern between different beams instead of a photomask. In order to resolve the diffraction limit of two methods, which deteriorates the resolution, we also introduce and review plasmonic-based interference lithography that is free from the diffraction limit. Each optical method has mutually complementary features in terms of the resolution, tunability, scalability, and compatibility.

The nanoimprint method has become an alternative to conventional photolithography for various applications for nanoscale devices. NIL transfers the pattern from the mask using direct contacting and resin curing. Therefore, it doesn’t require the expensive optical equipment including a shorter-wavelength light-source. In the case of NIL, we classify it into two smaller categories: conventional methods based on curing source, contact type and mold, and unconventional methods based on functional resin or resin-free processing. NIL that manufactures large-area metasurfaces with a fast speed is one of the practical fabrication methods. It has the capability to not only reuse the stamp but also realize high-resolution feature with the sophisticatedly carved mold. NIL can meet the requirements of fabrication of optical metasurfaces such as being high-resolution, low-cost, high-throughput, and achieving complexity for the three-dimensional (3D) structure. In the end of each section we review recent developments of different metadevices using the corresponding manufacturing methods.

## 2. Optical Methods

### 2.1. Conventional Photolithography

**Figure 1:**
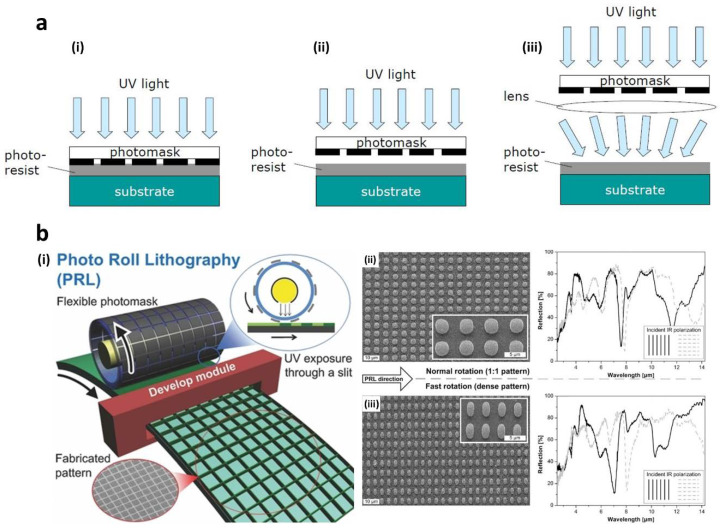
(**a**) Schematic of three typical methods of photolithography. (i) Contact, (ii) proximity, (iii) projection printing [64]. (**b**) (i) A schematic of the photo roll lithography (PRL) process. Scanning electron microscopy (SEM) images and infrared reflection spectra of (ii) round and (iii) oval Al dot arrays by the PRL using a same photomask at normal and fast mask rotating speeds [67]. Reproduced with permission from (**a**) [64]; Chulalongkorn University, 2012, and (**b**) [67]; WILEY-VCH Verlag GmbH and Co. KGaA, Weinheim, 2013.

Photolithography has been extensively utilized as a versatile micro/nano-fabrication technique that can be employed to fabricate large-scale metamaterials and devices in a short time [56,57,58,59,60]. The ability to make high-resolution and subwavelength structures has played a crucial role in the production of novel metamaterials that operate at various frequencies. Photolithography is defined as a process where light passes through a patterned photomask and is transferred to a photoresist (photo-sensitive chemical) below. The pattern is recorded in the photoresist on a substrate, and then the exposed or unexposed parts (depending on the kinds of photoresist, i.e. positive or negative) are selectively removed to make the desired polymeric structures. In this process, the photomask can be put in direct contact with or in close proximity to the photoresist layer (Figure 1a). The contact method puts the photomask in direct contact with the photoresist layer, while the proximity method uses a small gap between the photomask and the photoresist layer. The resolution of these photolithography systems is proportional to the wavelength and gap distance. Therefore, the contact method provides a higher resolution than the proximity method, since the gap is zero. However, damage and contamination of the sample and photomask can occur. This makes the contact method difficult to be applied to mass production. The proximity method can resolve such drawbacks as there is a non-zero gap providing the appropriate resolution, but the existence of a gap deteriorates the maximum achievable resolution due to near-field diffraction.

As mentioned above, optical metasurfaces that operate at visible frequencies require very high resolution down to hundreds of nanometers. Thanks to the continuous development of photolithography-related techniques, significant improvements in resolution have been achieved. The resolution, *R*, is defined as the minimal line width, which inherently suffers from the diffraction of light, and is expressed as follows:

(1) $$R=\frac{k_{1}\lambda }{NA}$$

where $k_{1}$ is an empiric coefficient with a limit of 0.25 depending on the photoresist, the pattern of the mask, and the optical system [61], λ is the wavelength of the exposed light, NA is the numerical aperture of the illumination system (expressed as nsinθ, where n is the refractive index of the medium and θ is incident angle of light). Contact and proximity printing can produce patterns down to a few micrometers [62], therefore cannot fulfill the sub-micrometer resolution required for optical metasurfaces that operate at visible frequencies. A number of techniques have been employed to achieve an increased resolution by manipulating the parameters in Equation (1). Projection printing has drawn great attention due to the ability to create high-resolution and large-scale arrays despite having a relatively small photomask, by using additional projection optics [62,63]. By projecting light through the same photomask repeatedly, large scale and high-throughput metasurfaces can be realized. By increasing the numerical aperture of the additional optical system, it can also achieve significant improvements in resolution. Moreover, the immersion method of using water as the background medium rather than air can also realize higher resolutions due to the increase of the refractive index, n [56,64,65]. Projection printing can be divided into two subsystems: steppers and scanners. Steppers only control the substrate, while scanners control both the photomask and the substrate simultaneously. Currently, the state-of-art projection printing primarily uses deep ultraviolet (DUV) and extreme ultraviolet (EUV) light with a wavelength of 193 nm and 13.5 nm, enabling to get sub-40 nm and sub-10 nm node resolution, respectively [66]. The introduction of DUV and EUV light satisfies the continuous demands of the semiconductor industry on high resolution features. For the reason mentioned above, projection printing using DUV and EUV has been actively utilized in the semiconductor industry. For example, Intel has established the 22 nm-chip manufacturing technology using the unique and advanced projection printing. This successful outcome of projection printing in the semiconductor industry is expected to lead to the practical metadevices.

Another attractive approach towards scalable fabrication is to integrate the rolling principle with the lithography-based methods. Photo roll lithography (PRL) utilizes controlled rolling of a hollow photomask-bearing quartz roller, inside which an ultraviolet (UV) source is mounted for continuous UV exposure of the photoresist-coated substrate fed underneath (Figure 1b). The flat photomask used in conventional photolithography is replaced by a flexible photomask that can be created on a common transparent film with improved scalability. As the rolling proceeds with the downward-collimated UV illumination through the slit (typically made on the UV source housing), the photoresist-coated substrate is continuously exposed to define the pattern. Notably, the resulting pattern shape can be modulated by relative motion control between the mask rolling speed and substrate feeding speed, which can be either elongated or shrunk compared to the original photomask pattern for optimizing the optical characteristics [67]. Likewise, phase shift lithography (PSL) can also be combined with the rolling principle. PSL is an optical patterning process that uses diffraction that occurs when light passes through a regular small pattern and uses a transparent mask of a specific shape to utilize optical diffraction in the near field [68]. Similar to PRL, a flexible roll-type phase-shift mask can be adopted to perform PSL in a continuous and scalable fashion.

**Figure 2:**
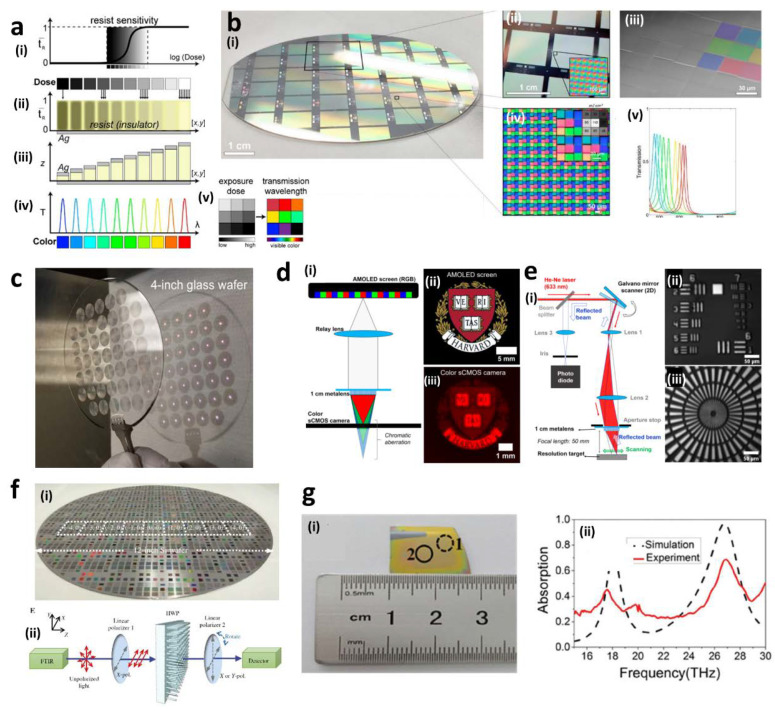
(**a**) Grayscale photolithography process of a multispectral filter array (MSFA) [69]. (i) Calculated profile of grayscale exposure dose, (ii) profile of a resist after exposure, (iii) the spatially variant three-dimensional (3D) resist profile after development and metal deposition, (iv) calculated transmittance spectra, and (v) spatially variant grayscale exposure dose matrix. (**b**) (i) Photographs of a 3 in MSFA, (ii) magnified (i) in a certain region, (iii) tilted SEM micrograph, (iv) optical micrograph of the different regions with each labeled identical exposure pattern (inset), and (v) the corresponding transmission spectra. (**c**) A tilted photograph of total 45 metalenses of 1 cm diameter on a 4 in a wafer [70]. (**d**) (i) Schematic of an active-matrix organic light-emitting diode (AMOLED) screen imaging, (ii) an image of Havard logo on the AMOLED screen, (iii) the measured image by a scientific complementary metal–oxide–semiconductor (sCMOS) camera using the fabricated metalens. (**e**) (i) Schematic of a reflective scanning microscopy using the metalens, (ii) the measured image of the United States Air Force resolution chart and (iii) a Siemens star using this reflective scanning microscopy. (**f**) (i) Photograph of a 12 in Si metasurface, (ii) schematic of a measurement system of Fourier-transform infrared (FT-IR) spectroscopy using the metasurface-based half-wave plate (HWP) [71]. (**g**) (i) Photograph of a centimeter-scale perfect absorber fabricated by photolithography, (ii) simulated and experimental spectra of the perfect absorber [74]. Reproduced with permission from (**a**,**b**) [69]; ACS Publications, 2019, (**c**–**e**) [70]; Copyright 2019 American Chemical Society, (**f**) [71]; De Gruyter, 2012, and (**g**) [74]; Royal Society of Chemistry, 2015.

In structural color filters, UV mask-based photolithography has been utilized to create large-scale color filter arrays. Many industries related with diagnostic medical imaging and remote sensing have been interested in structural color printing technology, but the absence of a scalable and inexpensive fabrication process has always been an obstacle that prevents commercialization. Mask-based photolithography can selectively control the size of each array using a spatially variant photomask. Spatially different 3D metal-insulator-metal cavity arrays could be realized by employing two grayscale lithography techniques (Figure 2a,b) [69]. Transmissive multispectral filter arrays (MSFAs) with low volumes generated through grayscale electron beam lithography (EBL) have been realized as proof-of-concept, and finally grayscale UV mask-based photolithography can be used to fabricate large-volume MSFAs on a 3 inch wafer scale in a single step. By utilizing the special photomask with the grayscale dose matrix, the resist thickness after development depends on the exposed dose determined by each grayscale pixel of the photomask. A resist with different thicknesses produces multispectral colored arrays. Therefore, tunable and selective multi-ranged spectra through grayscale photolithography can be realized on a single wafer.

Metalenses consist of subwavelength nanostructure arrays that induce a phase shift and are a highly promising alternative for traditional optics due to their ultrathin, lightweight, and tunable characteristics. Scaling nano-scale arrays up to the centimeter-scale has not been realized due to the absence of the corresponding manufacturing techniques. Recently, several groups have tried to make large-scale metalenses using photolithography. Among them, a centimeter-scale all-glass metalens was realized using DUV projection stepper lithography (Figure 2c–e) [70]. It can focus visible light and show diffraction-limited and polarization-insensitive focusing behavior. In addition, the ability for mass-production is proven by making 45 metalenses of 1 cm in diameter on a 4 inch wafer. The imaging capability of the 1 cm metalens is verified by using a direct imaging of an active-matrix organic light-emitting diode (AMOLED) display and reflective scanning microscopy. A collected image on an AMOLED screen by a relay lens is formed on a scientific complementary metal–oxide–semiconductor (sCMOS) camera after passing though the 1 cm metalens. Both measurement methods show consistent imaging results which agree with the focusing behavior results of the metalenses, and promising prospects of practical applications of metalenses made by photolithography.

Large-scale metasurfaces and metadevices realized through photolithography have also often played the role as a solution to the limitations of traditional optical devices. Waveplates are one of the most commonly used optical components and have broad applications in optical systems for polarization control. Existing optical waveplates require a large volume to accumulate the required phase differences for various polarizations. A metasurface-based half-wave plate (HWP) was fabricated with a 12 inch feature size by DUV photolithography (Figure 2f) [71]. The polarization conversion efficiency, which indicates the primary figure of merit for a HWP, is defined as $E_{c}=\frac{T_{cross}}{T_{cross}+T_{co}}$, where $T_{cross}$ and $T_{co}$ are cross- and co-polarized transmittances, respectively. The $E_{c}$ of the HWP was around 95% at the wavelength of 1.726 μm. Compared to other previous studies, the advanced fabrication process opens new way for mass production of metasurface-based waveplates and CMOS-compatible flat optics. Metasurface-based perfect absorbers are also required to be realized over large areas for practical applications such as photovoltaic devices, energy-harvesting devices, and stealth technology [72,73]. The dual-band perfect absorber showed absorption efficiencies of 45% and 75% at 18.1 and 26.8 THz, respectively (Figure 2g) [74]. Most importantly, the perfect absorber covered the whole 2 inch wafer using photolithography.

### 2.2. Interference Lithography (IL)

#### 2.2.1. Optics-Based IL

Laser-based manufacturing techniques have provided the required technological and cost-effective aspects to produce micro- and nanostructures using top-down approaches [75,76,77]. One technique of fabricating periodic structures is LIL [78,79,80,81]. In LIL, the interference pattern generated by the superposition of multiple laser beams is employed to expose a photoresist layer. When using a negative resist, the light-exposed portion is photo-polymerized. Consequently, periodic and polymeric structures can be generated after development. LIL has significant advantages compared with other existing techniques such as EBL and focused ion beam milling (FIB) in terms of large-area patterning, high- throughput, no contamination, low cost, and tunability. In addition, LIL does not require a photomask unlike photolithography.

Interference occurs when multiple electromagnetic waves overlap in the same space [82]. When such superposition of two or more waves occurs, the total electric field at a given position in space, r, can be expressed as the sum of all electric fields at that time, t:

(2) $$E\left(r,t\right)=E_{1}\left(r,t\right)+E_{2}\left(r,t\right)+\cdots +E_{n}\left(r,t\right)$$

where $E_{i}$ is the electric field of *i*th electromagnetic wave. The intensity distribution in the superposed region differs from point to point. The interference pattern depends on the phase difference between the electric fields. This means that the interference beams should be coherent, namely, they should be kept at a constant relative phase difference during exposure. For the experiment, the distribution of the intensity is recorded on a photoresist layer, and then after development the structures can be transferred onto a certain substrate through a sequent process.

The dimensionality of the generated periodic structures depends on the number of beams [83]. When the number of interfering beams, N, is below 4 and the difference of each wave vector is non-coplanar, the interference shows an (N−1) dimensional intensity grating. The electric field vector of each beam can be expressed with:

(3) $$E_{j}=E_{j}\hat{e_{j}}\exp\left[i\left(k_{j}\cdot r-\omega t+\varphi_{i}\right)\right]$$

where $E_{j}$, $\hat{e_{j}},$ and $\varphi_{i}$ are the amplitude of *j*th wave, unit polarization vector and phase, respectively. The intensity distribution on the photoresist layer, I, is expressed as:

(4) $$I=\underset{i}{\sum }E_{i}{}^{2}+2\overset{N}{\underset{i<j}{\sum }}E_{ij}{}^{2}{\hat{e}}_{ij}{}^{2}\cos\left[K_{ij}\cdot r+\varphi_{ij}\right]$$

where $E_{ij}{}^{2}=\left|E_{i}E_{j}\right|$, $K_{ij}=k_{i}-k_{j}$, $\varphi_{ij}=\varphi_{i}-\varphi_{j},$ and ${\hat{e}}_{ij}=\left|{\hat{e}}_{i}\cdot {\hat{e}}_{j}*\right|$. The differences of each wave vector, $K_{ij}$, determine the period of the interference fringe, and the contrast of that is adjusted by the amplitude of electric field, polarization, and phase of each beam [84]. If N is 2 that means only two beams are interfered, and a grating-like one-dimensional (1D) periodic intensity distribution is generated. Its period, d, also regarded as the resolution of the interference pattern, is determined by the wavelength of the incident light λ, the refractive index of photoresist n, and the incident angle of two beams θ. The pitch or resolution cannot surpass the value of d/2 due to the optical diffraction limit. d can be defined as:

(5) $$d=\frac{\lambda }{2n\cdot \sin\theta }$$

**Figure 3:**
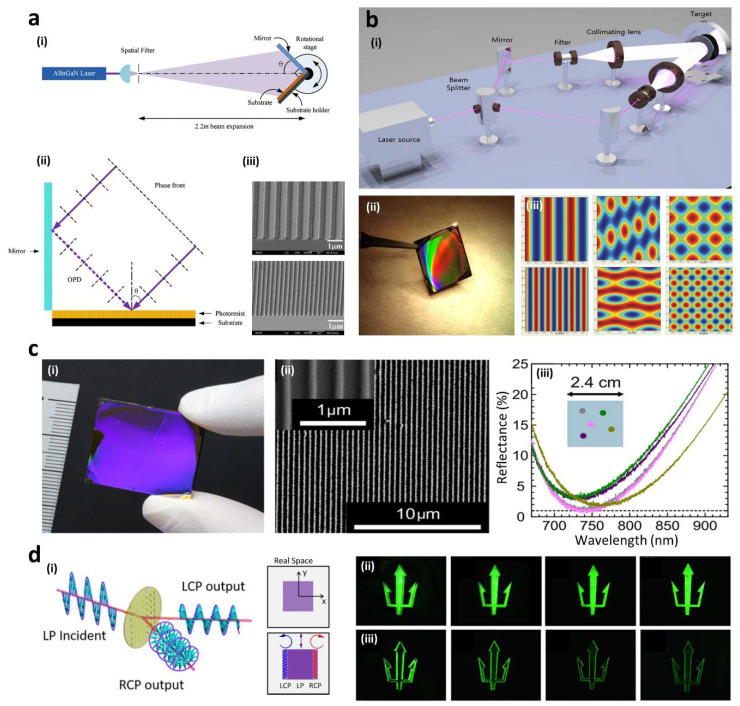
(**a**) (i) Schematic of a laser interference lithography (LIL) system using Lloyd’s mirror module, (ii) magnified schematic of (i) at the mirror and sample holder stage, (iii) SEM image of fabricated 1D gratings using the Lloyd’s mirror LIL: (top) 190 sec/750 nm and (bottom) 300 sec/290 nm of the exposure time/period, respectively [85]. (**b**) (i) Illustration of a LIL system using the beam splitter, (ii) photograph of a fabricated sample based on the LIL using beam splitter, (iii) different simulation results of interference using (left) two beams, (middle) three beams, and (right) four beams [90]. (**c**) (i) Photograph and (ii) SEM image of a spectrally selective perfect absorber using the LIL, (iii) measured reflectance spectra at 5 random positions [99]. (**d**) (i) Schematic of the edge detection concept based on a pre-designed phase gradient metasurface, (ii) measured images of two left- and right-circularly polarized (LCP and RCP) lights without the analyzer, playing a role as a reference, (iii) edge images corresponding to (ii). The resolution of pattern is 500, 750, 1000, and 8000 μm in both (ii) and (iii) from left to right, respectively [100]. Reproduced with permission from (**a**) [85]; IOP Publishing Ltd., 2010, (**b**) [90]; © 2013 IEEE, (**c**) [99]; Copyright 2016 American Chemical Society, and (**d**) [100]; United States National Academy of Sciences, 2019.

For the last few years, this laser-assisted interferometric method has proven not only the ability to fabricate different metasurfaces that requires subwavelength features but also the feasibility to realize practical applications based on its capability of mass-production. LIL can be separated into different methods according to the required optical modules. Lloyd’s mirror interferometer is the most commonly used method, and has a simple optical design consisting of a laser based on a coherent Gaussian beam, spatial filter (pinhole and objective lens), and a deflection mirror (Figure 3a) [85,86,87,88]. The mirror is located perpendicular to the sample holder that is an intersecting the position of two beams. Generally, the mirror and sample holder are fixed at 90 degrees and are placed on a rotation stage so that the period of the grating can be readily manipulated by varying incident angle θ in Equation (5). A beam from the laser is expanded by passing the spatial filter, and then the expanded or collimated beam reaches the photoresist layer on the substrate in two paths: one goes straight the substrate directly, and the other travels into the substrate after being reflected by the mirror. This optical path difference of the two beams causes the interference. At the same time, the coherence length should be long enough to surpass such an optical path difference. The Lloyd’s mirror interference lithographic tool has high tunability and easy manipulation for pitch control and a simple optical configuration, but the possible exposure area is limited by the size of the mirror. This limitation is minimized through another representative LIL system which uses two beams divided by a beam splitter (Figure 3b). Instead of a Lloyd’s mirror, the additional components of a beam splitter and a spatial filter generate two beam paths, thereby the exposure area is not limited. In this configuration, it is possible to increase the complexity of the target structures by utilizing more than two beams in order to generate a more complex interference pattern [89,90,91,92]. If the laser has sufficiently high energy like a pulsed laser, the materials can be directly patterned with direct irradiation. This advanced method has been called direct laser interference patterning (DLIP) [93,94,95]. Similar to the original LIL method, the radiation of the laser enables different metallurgical processes such as melting, crystallization, or recrystallization of amorphous materials. This area was actively studied with silicon in the 1990s [96,97,98].

A perfect absorber operating at visible to near-infrared wavelengths was produced by Lloyd’s mirror interference lithography in order for spectrally selective perfect absorption to be realized with simple control of the structural pitch (Figure 3c) [99]. The absorption frequencies of the palladium-based perfect absorber depend on the shape and size of the structures. The 1D grating-based device shows nearly perfect absorption at the resonance wavelength of 740 nm, while a two-dimensional (2D) square-based device also shows nearly perfect absorption at the resonance wavelength of 950 nm.

In an optical edge detection device, femtosecond DLIP was used to fabricate metasurface patterns inside glass (Figure 3d) [100]. Optical edge detection is a fundamental method in image processing, and significantly reduces the amount of data to be processed because it extracts important information and preserves meaningful geometric features. The device showed optical efficiency around 90%. For strong laser irradiation, a plasma of high free electron density occurs by a multiphonon ionization process. The interference of such plasma and the incident light creates the rectangular nanostructures. The desired direction of the nanostructure perpendicular to the polarization was achieved by manipulating the polarization of the incident light.

#### 2.2.2. Plasmonic-Based Interference Lithography

As mentioned earlier, the resolution of LIL using existing optics cannot theoretically surpass d/2 due to the optical diffraction limit. A possible way to improve its resolution is to use light with significantly shorter wavelengths such as DUV, EUV, and soft x-rays, but the cost of setup will become extremely high. Plasmonic lithography is a promising candidate as a future nanofabrication tool. This is a photon-based technology which can resolve space-charge and serial writing limitations and can overcome the optical diffraction limit of Equation (5). When electromagnetic waves interact with free electrons on the surface of a thin-film metal or subwavelength structure, oscillation of the electrons is induced by the incident wave. The collective oscillation of electrons is known as surface plasmons (SPs) which exhibit unique optical behaviors [101,102]. The propagating waves due to SPs along the metal-dielectric interface are known as surface plasmon polaritons (SPPs). The wave vector of SPPs can be defined as:

(6) $$k_{sp}=\frac{2\pi }{\lambda_{0}}\sqrt{\frac{\varepsilon_{d}\cdot \varepsilon_{m}}{\varepsilon_{d}+\varepsilon_{m}}}$$

where $\lambda_{0}$ is the wavelength of light in vacuum, and $\varepsilon_{m}$ and $\varepsilon_{d}$ are the permittivities of the metal and dielectric, respectively. The dispersion relation of Equation (6) can also be expressed as $\lambda_{sp}=2\pi /k_{sp}$ where $\lambda_{sp}$ is the wavelength of SPs. The $\lambda_{sp}$ is much shorter than $\lambda_{0}$, especially near the resonance condition where Re(εd)=−Re(εm), indicating the real parts of $\varepsilon_{d}$ and $\varepsilon_{m}$, respectively. The opposite values infer subdiffractional resolution imaging capabilities. Most importantly, thanks to the extremely short wavelengths of SPPs, deep-subwavelength nanofabrication beyond the optical diffraction limit up to the resolution of λ/8 can be actualized.

**Figure 4:**
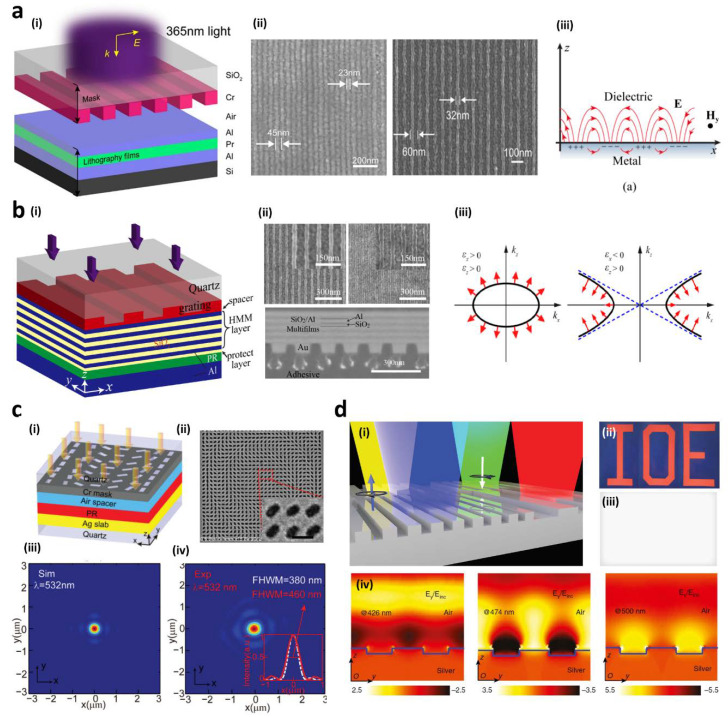
(**a**) (i) Schematic of a structure of the surface plasmon interference lithography (SPIL), (ii) SEM images of the patterned grating results with 22 and 32 nm half-pitches [129], (iii) schematic of surface plasmon polaritons (SPPs) on dielectric-metal interface [130]. (**b**) (i) Schematic of a structure of surface plasmon polaritons (BPPs) interference lithography with hyperbolic metamaterials (HMMs), (ii) SEM images of the patterned grating results of (left) titanium dioxide, (right) gold and (bottom) cross section of the BPPs structure, respectively [114], (iii) the dispersion relations of (left) natural materials and (right) HMMs. The group velocity is indicated by the arrows [130]. (**c**) (i) Schematic of a structure of the surface plasmon imaging lithography, (ii) SEM image of the fabricated metalens (scale bar, 200 nm), (iii and iv) the cross section intensity distribution for circularly polarized light with 532 nm wavelength, (iii) the simulated and (iv) experimental results [115]. (**d**) (i) Schematic of a structural coloring device using the plasmonic lithography structure, (ii and iii) microscopic images of a metasurface indicating the letters “IOE” with (ii) 45°/−45° and (iii) 45°/45°, respectively, (iv) the maps of electric field intensity distribution with a period of the wavelength of 400 nm at 426, 474, and 500 nm from left to right, respectively [128]. Reproduced with permission from (**a**–**i**, **ii**) [129]; Copyright 2019 American Chemical Society, (**b**–**i**, **ii**) [114]; OSA Publishing, 2018, (**a**–**iii**, **b**–**iii**) [130]; MDPI, 2016, (**c**) [115]; Royal Society of Chemistry, 2015, and (**d**) [128]; De Gruyter, 2017.

In 2004, a 1D aluminum (Al) grating of 300 nm pitch and 50 nm width was used to successfully excite SPPs with 436 nm incident light. A interference pattern of 100 nm pitch was transferred onto a resist [103]. Since this first report, numerous studies of plasmonic lithography using SPPs have conducted, and the technique has been termed surface plasmon interference lithography (SPIL) (Figure 4a) [104,105,106,107,108]. Recently, in order to enhance and improve the capabilities of SPIL, an advanced design was presented, so-called bulk plasmon polariton (BPPs) interference lithography (Figure 4b) [109,110,111]. BPPs with large wave vectors propagate into pre-designed bulk hyperbolic metamaterials (HMMs) [111]. HMMs composed of alternatively deposited metal/dielectric multilayers exhibit a hyperbolic dispersion instead of elliptical dispersion, allowing for a larger-area and uniform deep subwavelength pattern to be generated inside a photoresist [34,112]. So far, 45 nm and 25 nm resolutions of half-pitch ( λ/8 and λ/16) have been fabricated and simulated with different interference patterns, respectively [113]. Furthermore, a 35 nm resolution of half-pitch ( λ/10) was fabricated [114].

For metalenses, a novel high-throughput nanofabrication method was realized by employing plasmonic imaging lithography (Figure 4c) [115]. The metasurface with anisotropically arranged nano-slots was made with further single-layer and multi-layer transfer etching steps. The metalens exhibited great focusing performance in optical measurements. This plasmonic lithography provides many benefits such as high-resolution patterning and low cost owing to the commercially available photoresist and light source. For these reasons, it is a promising candidate to make numerous metasurfaces and metadevices such as structural color filters [11,116,117,118], perfect absorbers [119,120,121], beam modulators [122,123,124], metaholograms [18,125,126,127], and so on.

Plasmonic lithography was used to realize a large scale plasmonic coloring device (Figure 4d) [128]. The device exhibited high efficiency up to 75% at different wavelengths, and highly decreased efficiency at off-resonance wavelengths. The results show spin-restored colors generated by sharp resonances. In the fabricated plasmonic grating, the electric field is strongly enhanced at the silver-air interface at wavelengths of 426 and 474 nm. Such strong electric field enhancement is resulted from the collective oscillation of the free electrons, i.e., SPPs, at the silver-air interface. On the other hand, the electric field is locally enhanced at the sidewalls and corners of the metal groove. This result occurs because both SPPs and localized SPs are functioned to induce the phase difference of 180° between each perpendicularly linear polarization components. This fact is proven in Figure 4d(ii) and Figure 4d(iii) where all colors disappear when the incident and reflective lights exhibit the same polarization state. This intriguing phenomenon presents the promising possibility for security and encryption displays.

## 3. Printing Methods

**Figure 5:**
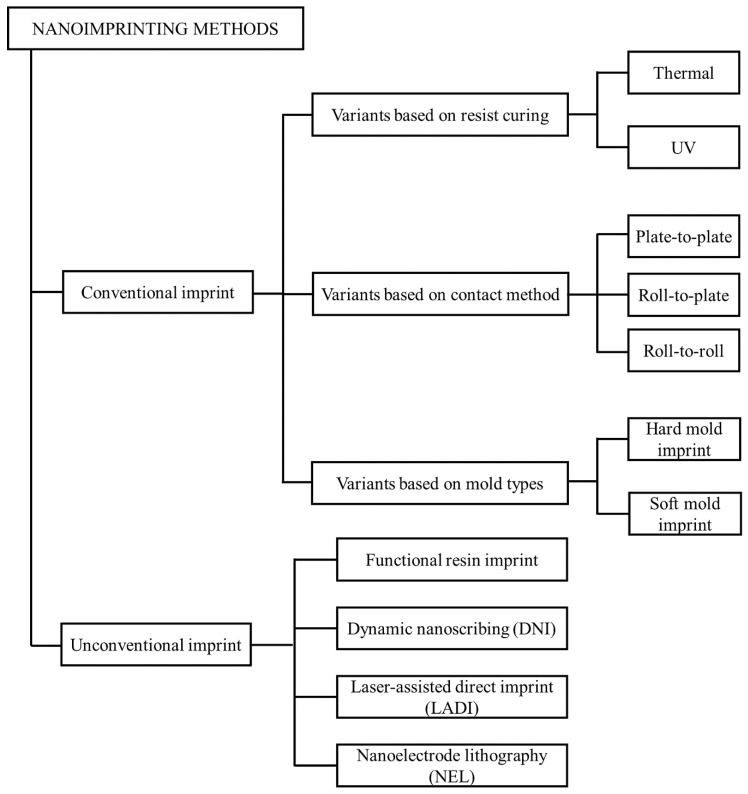
Classification of nanoimprinting methods. Conventional imprint methods indicate the general classification criteria of nanoimprint lithography (NIL) methods including curing methods, contact methods, and mold types. Unconventional imprinting contains the emerging methods which are hard to classify using the conventional criteria.

Early NIL methods used thermal heating and a UV light source as resin curing sources. It is used in industry and has been studied actively. We called those methods conventional nanoimprinting. However, there are several emerging methods including functional resin-based printing (e.g., sol-gel process and nanoparticle resin) and resin-free imprinting (e.g., laser process and direct inscribing), here referred to as non-conventional nanoimprinting. Particularly, conventional printing methods have various variant criteria such as curing sources, contact methods, and mold types as shown in Figure 5. We introduce the basic principle and metasurface applications for each method in the following sections.

### 3.1. Conventional Printing Methods

#### 3.1.1. Curing Source

**Thermal NIL** In 1995, Choi et al. invented the NIL method, which can be used to fabricate nanoscale patterns to a sub 25 nm resolution with a 100 nm depth [131,132]. Mold-based methods need filling and solidification. This method, which based on heating and cooling, is used for filling and curing and was named thermal NIL. In 1966, Haisma et al. introduced UV based NIL which uses UV the curing source [133]. This method can maximize productivity by eliminating the heating and cooling processes. A comparison between thermal and UV NIL is shown in Table 1.

**Figure 6:**
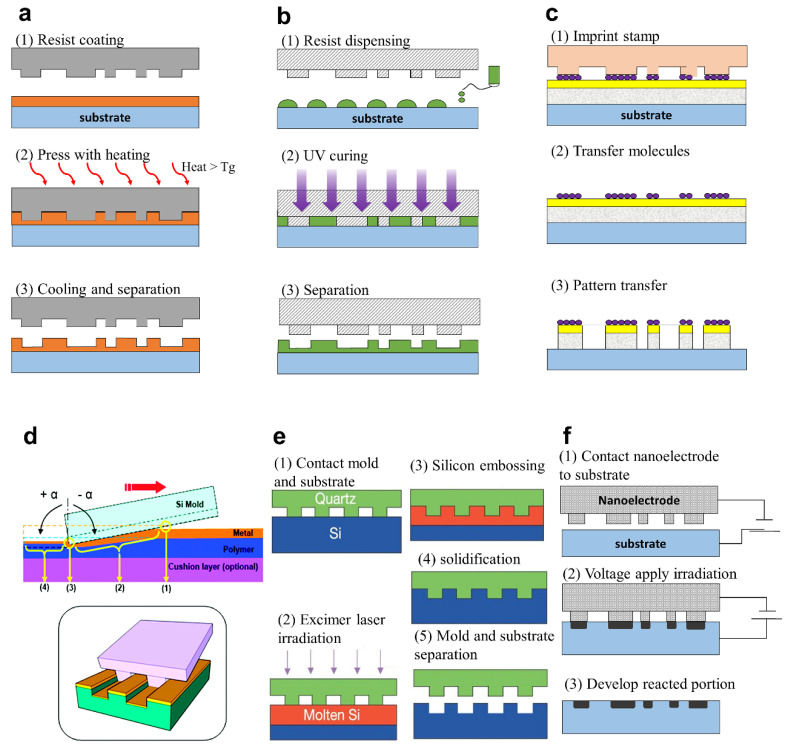
Schematics of various NIL processes: (**a**) Thermal NIL, (**b**) UV NIL. (**c**) Soft lithography. (**d**) Dynamic nanoinscribing (DNI): (1) initial contacting point, (2) gradual imprinting region, (3) edge point responsible for plastic deformation, and (4) elastic recovery region [136]. (**e**) Laser-assisted direct imprint (LADI). (**f**) Nanoelectrode lithography (NEL) [137]. Reproduced with permission from (**d**) [136]; American Chemical Society, 2009, and (**f**) [137]; American Vaccum Society, 2003.

A schematic of thermal NIL is shown in Figure 6a. Thermal NIL uses the resist with low viscosity at high temperature (i.e., above the glass transition temperature, T_g_). Most resists require spin coating due to their high viscosity at low temperatures. After heating above the T_g_, which depends on the resist material (usually 100–200 °C), pressure (usually 300–1900 psi) is applied to fill the nanoscale patterns in the mask [134]. However, air bubbles can lead to under filling. This is solved by using a vacuum. The appropriate process temperature is generally 50–70 °C higher and 20 °C lower than transition temperature [135].

Thermal NIL has several challenging issues such as determining the proper resist and demolding. The processing procedures, including the pressure time and mold temperature, are dependent on the material of resist. The resist also experiences conformal bending over a wide area which causes flexion of the mask and substrate [138]. Another main block of thermal NIL is the stress from the demolding step due to heat expansion. Recent research has used piezo-driven vibrations to reduce the contact stress [139]. Novel thermoplastic (modified mr-I700R) has also been used to alleviate the pull off force [140].

**Figure 7:**
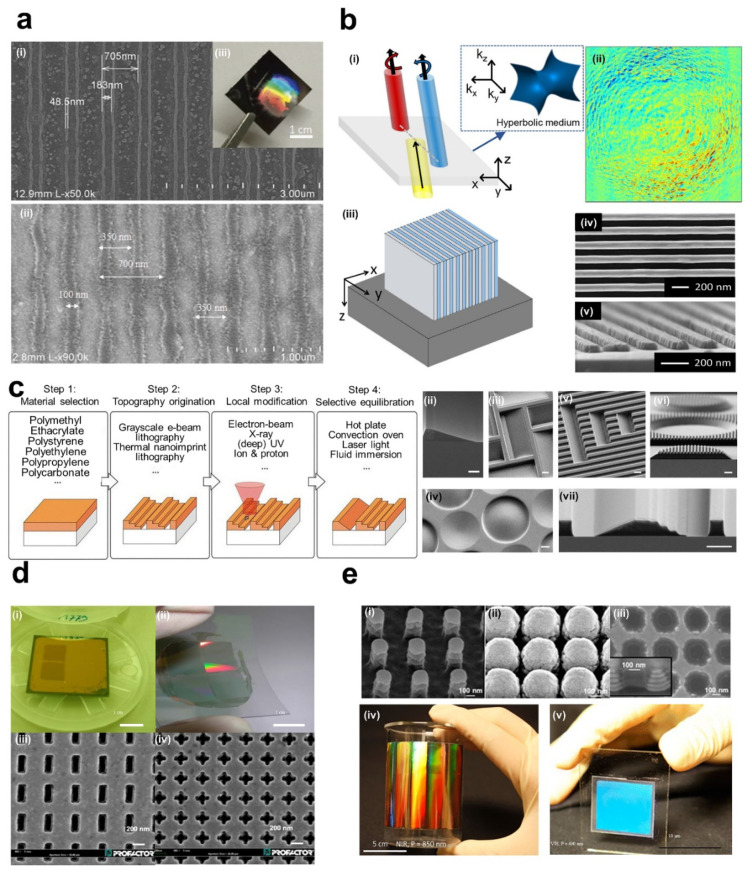
Thermal UV NIL (**a**) (i) SEM image of periodic photoresist pattern by the bare AI grating mask, (ii) pattern by HMMs multilayer, (iii) a photograph of the produced photoresist grating on the polyethylene terephthalate sheet [104]. (**b**) Vertical HMMs fabrication and its optical spin Hall effect. (i) Schematics of optical spin Hall effect in real space, (ii) a measured result of the distribution of transmitted beam. The red and blue colors indicate the LCP and RCP lights, respectively, (iii) schematics of vertical HMMs, the SEM image of (iv) top view and (v) perspective view [34]. (**c**) (i) Schematic flow sequence of the general taste method for the generation of asymmetric 3D grating pattern, (ii–vii) the SEM micrographs of the refined poly methyl methacrylate (PMMA) topographies after exposure of pre-patterned PMMA with high-energy electrons and subsequent thermal annealing (scale bars: 1 μm) [142]. (**d**) (i) Photograph of a single-layered metasurface sample, (ii) transfer-printed gold grating on a flexible foil (iii) SEM image of top view, (iv) SEM image of Swiss-cross metamaterials [155]. (**e**) A 3D negative index metamaterial which has telecom band with the large-area. (i) Top-viewed SEM image before the deposition of NIL with the 850 nm period, (ii) after deposition of 11 Ag/MgF_2_ layers, (iii) after removal of polymer posts (inset image shows the tilted view), the photographs of the fabricated results on (iv) the flexible and (v) the rigid substrate [156]. Reproduced with permission from (**a**) [104]; Japan Society of Applied Physics, 2015, (**b**) [34]; American Chemical Society, 2019, (**c**) [142]; CC BY 4.0, (**d**) [155]; IOP Publishing Ltd., 2011, and (**e**) [156]; American Chemical Society, 2014.

At the laboratory scale, thermal NIL is widely used to fabricate the metamaterials because the equipment is much simpler and cheaper than UV NIL. Two-step thermal NIL was developed to fabricate large-area plasmonic metamaterials with giant chiroptical responses [141]. The first NIL process was used to make a negative Al mold for the second NIL process which can make more than 3 cm^2^ area plasmonic resonance structure. Jay et al. used thermal NIL to fabricate a 3D structure with a 100 nm linewidth and 350 nm periodicity over a 1.5 × 1.5 cm^2^ area (Figure 7a) [104]. Multilayer structures can be used for metamaterials, suggesting a cost-effective and less laborious technique for large-area nanopatterning. Kim et al. demonstrated a vertical HMMs to enhance optical spin Hall effects fabricated by thermal NIL (Figure 7b) [34]. The enhanced Hall effect of the hyperbolic metasurface enables helicity-dependent control for optical devices such as sensors, switches, and filters. A novel 3D nanofabrication method that combines several pre-developed methods called thermally activated selective topography equilibration (TASTE) was developed more recently [142]. It includes thermal NIL, EBL, and laser light (Figure 7c).

**UV NIL.** To overcome the time and temperature limitations of thermal NIL, UV NIL was introduced. It is a direct contact molding process that can replicate the pattern from a mask using a photocurable resist and UV exposure at room temperature (Figure 6b). After the first development of UV NIL [133], several influential technical improvements in terms of the resolution, tools, and resist design were established [143,144]. Low-viscosity resists were arranged by the inkjet printer head depending on the density of the pattern. The mask, which is aligned to the substrate, moves down to the resist, and resist flows into the mask due to the capillary force. Next, UV light expose to the resist through transparent mask. After removing the mask, the solidified resist pattern is printed onto the substrate.

There are several building blocks for UV NIL in respect of imprint materials, and masks [145]. The various properties of the imprint material need to be considered, such as the fluid (dispensability, viscosity, evaporation, wettability), and mechanical properties (yield strength, adhesion force with substrate, release stress) [146]. Low-viscosity materials are generally preferred to achieve high filling speed. However, low-viscosity can lead to the evaporation, which can increase the process cost [147]. Therefore, the balanced selection of proper viscosity and evaporation is necessary. For the mask or mold of NIL, silica or quartz are mainly used because mask should be transparent for UV curing. However, those materials are non-conductive so a charging effect is caused. It distorts the electron beam and affects the fidelity and resolution. To preventing such effects, a thin metal layer (e.g., chromium 15 nm) or conductive oxide layer (e.g., indium tin oxide) are used. The high-resolution mask fabrication is also essential for nanoscale patterns. Michel et al. build the 6 nm half pitch pattern with the specially prepared mold [148]. It uses the silicon dioxide based on the electron beam with polystyrene. For the complex 3D profile, FIB directly carves out the mask for NIL [149,150,151]

There have been several attempts to use NIL to create metamaterials over a large-area quickly [152,153]. Wu et al. firstly developed a metasurface that worked at near-infrared wavelengths with L shaped components made using NIL. After that, highly nonlinear optical spectroscopy metasurfaces were also developed using UV NIL [154]. Moreover, 3D structures were designed using NIL. Through the fast, room temperature process, several single layers were easily built to create the 3D structure to work at the wavelength of 1.8 μm (Figure 7d) [155]. Furthermore, Gao et al. created a large-area (>75 cm^2^) 3D metamaterial (fishnet base) that works in the visible band as shown in Figure 7e [156].

#### 3.1.2. Contact Type

**Figure 8:**
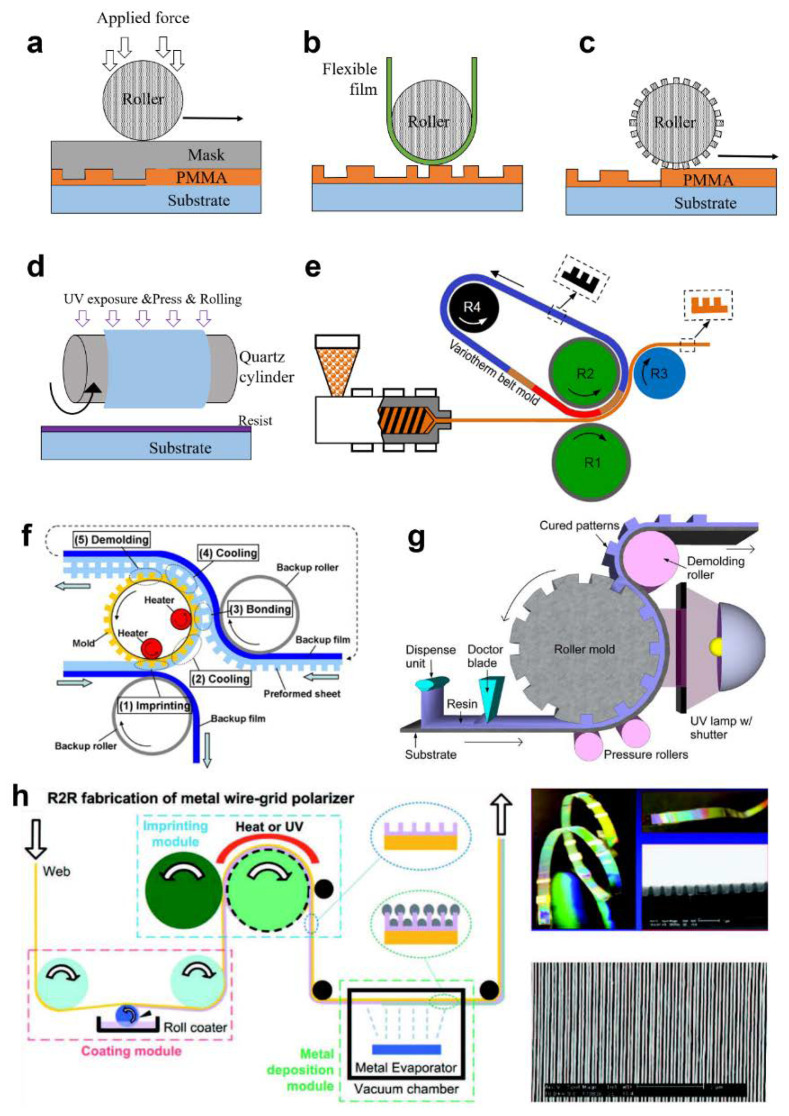
Schematics of roller-based NIL. (**a**) Roll to plate (R2P) NIL using a flat mold with a roller press. (**b**) Thermal R2P NIL systems using a flexible polymer film. (**c**) Thermal R2P concept with pattern stamper. (**d**) UV R2P NIL using a flexible mold. (**e**) Thermal R2R process with a variotherm belt mold [164]. (**f**) Thermal R2R NIL system for multilayered structures [166]. (**g**) Typical UV R2R NIL system [167]. (**h**) A schematic of the fabrication flow of the metal wire grid polarizer using UV R2R NIL [168]. Reproduced with permission from (**e**) [164]; CC by 4.0, (**f**) [166]; Elsevier B.V, 2009, (**g**) [167]; AIP Publishing, 2012, and (**h**) [168]; WILEY-VCH Verlag GmbH and Co. KGaA, Weinheim, 2008.

NIL can be divided into three contact methods: plate-to-plate (P2P), roll-to-plate (R2P), and roll-to-roll (R2R). It is a challenge for P2P NIL to produce large-area structures up to the wafer-level (~300 mm). Overall conformal contact occurs that causes a non-uniform pressure distribution. Moreover, surface to surface contact needs higher pressure than line to surface contact. Those higher pressures not only require complex equipment, but also reduce the resolution of the pattern (Table 2). The first attempt of roller based NIL directly applied the force using the roller as in Figure 8a. This method has similar throughput to the P2P method, however, it can reduce the total required force by changing the surface contact to a line contact. P2P based 8 inch wafers can take 20,000 N of imprint force [157], however, the R2P method only needs 200 N for a 300 mm width imprint. Flexible polymer film based R2P methods were also introduced as shown in Figure 8b [158,159]. They used the thermal NIL mechanism with a heated mold and roller. Another R2P application used a flexible mold which wrapped by the heated roller, not a flat mold (Figure 8c) [160]. Park et al. combined the UV NIL units and R2P methods and also used a flexible mold but with UV as the curing source (Figure 8d) [161]. This system can fabricate 70 nm line width patterns and 30 nm dot array patterns at room temperature. Uniform and scalable resin coating tactics are essential for large-area NIL. For instance, simple spin-coating or drop dispensing is not suitable for high-speed continuous process based on R2R system. Airbrushing can provide one promising solution for continuous and uniform resin coating over a large-area substrate, prior to feeding it to the mold-contacting zone in UV NIL. Koo et al. demonstrated the uniform airbrushing of UV-curable resin on a substrate that was continuously fed to the R2R UV NIL [162]. Controlling the resin concentration and airbrushing conditions can enable residual layer-controlled R2R NIL in a continuous manner without resorting to spin-coating or other non-continuous resin coating methods [163].

Continuous processes are impossible for R2P based methods because roller lifting and returning steps are essential. Therefore, several research groups have applied R2R methods. R2R NIL uses flexible substrates and a supporting roller. It can realize industry level throughput. As shown in Figure 8e, a new extrusion thermal R2R imprinting with a variotherm belt mold was introduced [164]. The components of this system include an R2R setup, a variotherm belt, and an extruder with a heating unit. As a result, 30 µm sawtooth patterns at a speed of 10 m/min have been produced. Mäkelä et al. built the thermal based R2R NIL [165]. It combines the gravure unit and a nanoimprinting unit with the conducting polymer. For advanced nanodevices such as metasurfaces, 3D and multi-layered structures are essential. Nagato et al. suggested iterative roller imprint for multi-layered nanostructures using both bonding and thermal R2R NIL [166]. As shown in Figure 8f, the first layer is imprinted by heated rollers. Next, the thin film is attached to the backside of the imprinted layer with other rollers. They fabricated a 300 nm deep 800 nm pitch nanograting. Figure 8g shows the UV R2R NIL system. It consists of a dispensing unit, a doctor blade, a roller mold, pressure rollers, a demolding roller, and a UV source unit [167]. First the dispensing unit deposits a UV curable resin onto the continuous film. The doctor blade controls the thickness of the liquid resin. After that, the multi roller system applies pressure to the roller mold with UV exposure. In the last step, the demolding roller peels off the curved pattern from the roller mold. UV R2R NIL is complex but enables the continuous fabrication for mass production. Ahn et al. developed an R2R system which operates through UV based NIL (Figure 8h) [168]. It can produce 80 nm line structures with a 1400 mm/min throughput. As an alternative technique to realize scalable UV NIL without rollers, Ok et al. used both the liquid and solid state of the UV resin to continuously inscribe the substrate through the edges of a grating-patterned mold and cure the resin at the same time [169]. They also applied the process to various flexible substrates, and easily produced patterns with a resolution of about 100 nm.

**Figure 9:**
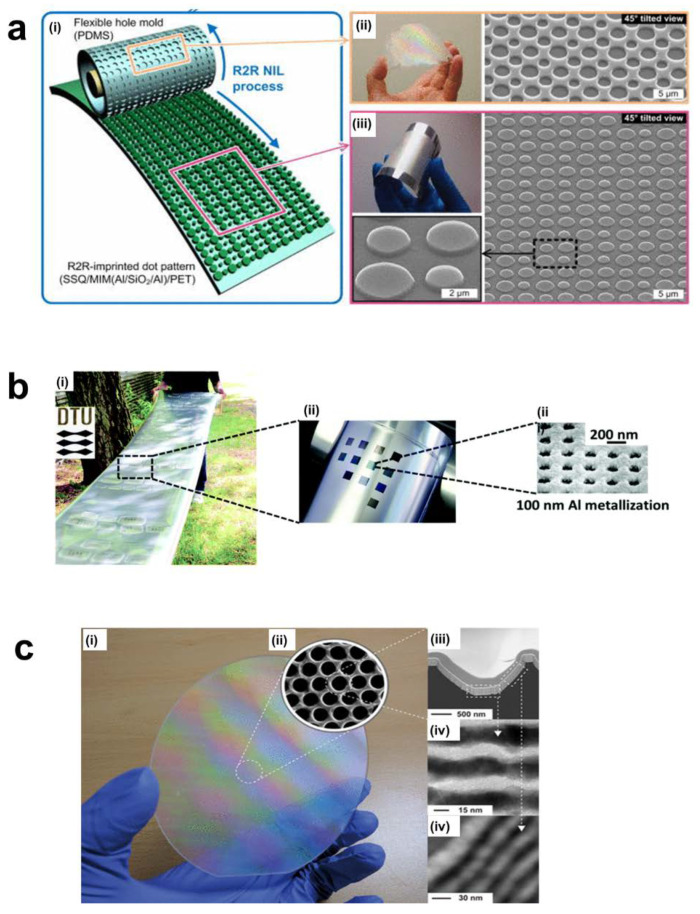
(**a**) (i) Illustration of the overall fabrication of R2R imprint based on the flexible polydimethylsiloxane (PDMS) mold, (ii) rolling over a substrate under conformal contact, (iii) imprinting sot pattern with a very thin residual [171]. (**b**) A large-area plasmonic coloring metasurface using high-speed R2R method, (i) a polymer foil structure with 100 nm Al including the university logo fabricated by plasmonic color printing (1 × 1.5 cm), (ii) the magnified image of a plasmonic color patch, (iii) the SEM image of the patch with 100 nm metallization [173]. (**c**) (i) Photograph of a fabricated hyperlens product on a 4 inch wafer size. The SEM images of (ii) top view, (iii–v) The transmission electron microscopy (TEM) images of the single hyperlens with various magnification at (iv) center area and (v) side-wall indicate 15 nm thick layers [4]. Reproduced with permission from (**a**) [171]; AIP Publishing, 2012, (**b**) [173]; Royal Society of Chemistry, 2017, and (**c**) [4]; American Chemical Society, 2018.

The research and development phase of metamaterials is still mostly at the laboratory level. However, several research teams have attempted roller based NIL to create metamaterials [170]. Ok et al. developed the first metamaterial films through the R2R method [171], a dual-band infrared (IR) filter for the 6–8 and 10–12 μm wavelengths (Figure 9a). Rai et al. suggested the schematic for an R2R NIL based fishnet that was already developed by EBL [172]. Plasmonic color metasurfaces have also been fabricated using high-speed (10 m/min) UV R2R NIL (Figure 9b) [173]. This high-throughput manufacturing method paves the way for the extension of metasurface applications such as printing, memory, and biosensors. However, until now, roller-based methods still have imitations such as thermal expansion (thermal roller NIL), mold sticking, and mold lifespan that prevent stable mass production.

#### 3.1.3. Mold Type

The first NIL only used hard masks, but flexible masks were introduced to apply R2R NIL methods. This can improve the durability, chemical resistance, biocompatibility, and price competitiveness. Lithography with soft (flexible) molds (Figure 8c), also called as soft NIL, involves soft embossing, imprinting, pattern transfer molding, and capillary molding [174]. It can be conducted outside of the clean room at a low cost and with a facile process. Si et al. made a large area (25 cm^2^) device in the ambient atmosphere using polydimethylsiloxane (PDMS) molds [175]. This overcomes the challenges caused by the oxygen-containing atmosphere. This research demonstrated that soft NIL methods can be used to reduce the cost of fabrication and can easily be adapted to large, curved surface structures. Bhingardive et al. overcame the soft NIL resolution limitation by making a sub-100 nm feature size nanopattern based on soft thermal NIL [176]. Despite its many advantages, however, soft NIL still has the fundamental limitation of resolution [177].

Byun et al. demonstrated the possibility of hyperlens fabrication beyond the diffraction limit using a flexible PDMS mask and UV NIL [5]. As a follow-up study, Lee et al. realized a wafer-scale hyperlens (Figure 9c) which can observe biomolecular images in real-time (>151 nm) [4]. NIL was also applied to make various types of light emitting diodes (LED). A biosensor based on localized surface plasmon resonance (LSPR) was also developed using soft UV nanoimprint over a large surface [178]. It showed perfect omnidirectional broadband absorption in the IR region with high refractive index sensitivity, more than 10 times higher than conventional LSPR. Wi et al. proposed a continuous production method of LSPR-based sensors on a transparent flexible substrate by integrating a soft R2R NIL process and an angled evaporation process. They also measured the beta amyloid to the femtogram-level (10^−15^ g) using the fabricated sensor, and verified the excellent LSPR sensor performance [179]. As a follow-up study, they used the LSPR sensor to measure the concentration of polystyrene beads by size, showing the versatility of the complex 3D plasmonic nanostructure [180].

### 3.2. Unconventional Printing Methods

**Figure 10:**
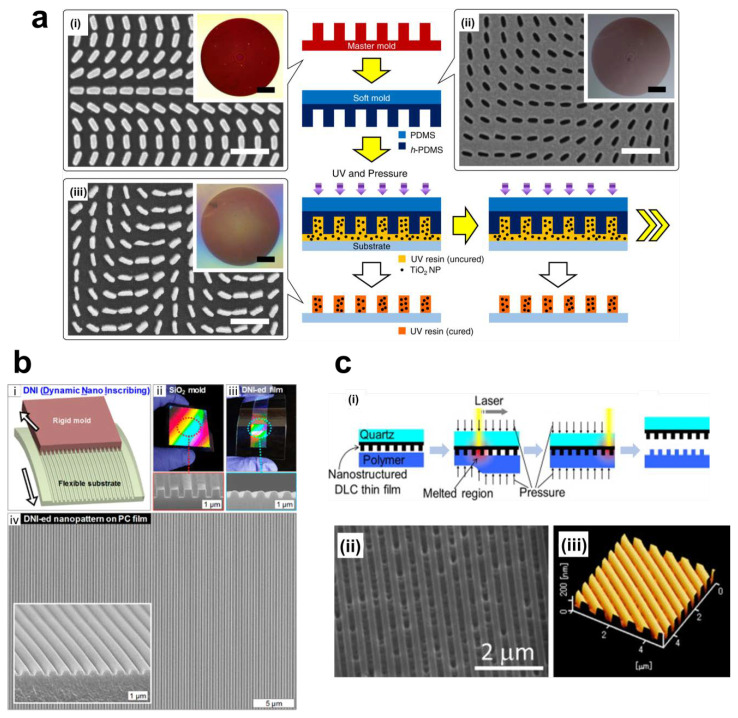
(**a**) Schematic of the plum pudding metasurfaces. Titanium dioxide nanoparticle composite resin is cured by PDMS and hard PDMS molds. The SEM images of (i) master mold, (ii) soft mold, and (iii) final metalens. All scale bars are 100 μm [2]. (**b**) (i) Mechanism of the DNI process, (ii) a rigid nanograting mold, (iii) a inscribed nanopattern on a flexible substrate, (iv) the SEM image of a nanopattern using the DNI. Inset indicates the enlarged perspective view [185]. (**c**) Laser-assisted large-area nanoimprint method and its results. (i) Schematics of laser-scanning replication, (ii) the SEM image and (iii) the atomic force microscope profile of the mold [192]. Reproduced with permission from (**a**) [2]; CC by 4.0, (**b**) [185]; American Chemical Society, 2019, and (**c**) [192]; Elsevier B.V., 2014.

Conventional imprinting methods only use the typical resins based on thermal and UV NIL. However, to improve the functional ability and price competitiveness, various resin and coating methods have been explored. We called it as unconventional printing methods. It includes not only functional resin based NIL, but also covers the resin-free imprint. Checcucci et al. applied a functional resin which consist of titanium sol-gel coating to fabricate dielectric resonators [181]. By applying the titania coating, they can make the three orders of magnitude patterned size, and three times smaller squared pillars respect to their previous report [182]. The porous titania resonators have multiple applications including tuning the sharp bandpass filter (up to 40 dB rejection ratio), gas or liquid sensor, and dynamic color changing structure. To achieve both the low cost and high refractive index nanopattern, Yoon and Kim et al. suggest the facile metamaterial manufacturing process based on the resin with the titania nanoparticles [2,171]. Combining the UV-curable resin and nanoparticles can increase the refractive index without secondary operation (Figure 10a).

In addition to imprinting the resin with a light source such as UV, direct imprinting processes with various energy sources (heat, laser, voltage) without a resin are actively being studied. Among them, heat energy is the most readily available. Scanning probe lithography (SPL) processes can fabricate very precise nanopatterns from 1 to 100 nm by imprinting a sub 10 nm tip directly on the substrate [183]. Among many SPL processes, high-speed thermal scanning probe lithography (t-SPL) process is capable of sub 10 nm nanopatterning over an area of 880 × 880 pixels in 12.8 s [184]. This process induces a depolymerization reaction on the surface of the polymer substrates by raising the temperature of the tip to about 700 °C to produce sophisticated nanopatterns. However, the biggest limitation of this process is difficulty of initial setup and the durability of the tip.

To supplement the limitations of SPL, Ahn et al. introduced the process of directly inscribing nanograting patterns on flexible substrates using the sharp edge of a patterned mold, called dynamic nanoinscribing (DNI), as shown in Figure 6d and Figure 10b [136]. DNI is a continuous process in the existing hot embossing process and is drastically simplified by replacing the face-to-face process with a line-to-face mechanism. In addition, the t-SPL process shows the depolymerization on the surface of substrates at the process temperature of about 700 °C, whereas the DNI process engraves the pattern at the polymer’s T_g_ (about 100 °C), thus it only physically fabricates nanopatterns without chemical reaction. It can be applied to a variety of metals and polymers to create patterns with a sub-50 nm size at very high-speed (10 cm/sec). As a follow-up study, Oh et al. advanced the DNI process by revealing the correlation between the pressure and temperature of the process and controlling the shape, depth, and dimensions of a nanopattern as shown in Figure 10b [185]. Vibrational indentation patterning (VIP) has a similar mechanism and characteristics to DNI but it does not require thermal energy [186]. It uses the vertical vibration of a flat and sharp edge, which can make periodic indentations on the substrate. Moreover, Ok et al. combined the DNI and VIP process to make 2D nanostructures with two sequential 1D processes with a very simple mechanism [187]. With the advantages of both DNI and VIP methods such as the compact and vacuum-free system, it is competitive imprint method for precise machining for optical applications [145,188]. For example, Oh et al. developed flexible reattachable ionomer nonpatterns (RAINs) with DNI and low-temperature roll imprinting [189]. They can be fabricated under reasonable temperatures as low as 60–70 °C, so can therefore be used for thermosensitive products such as organic light emitting diodes (OLED).

As shown in Figure 6e, Chou et al. developed laser-assisted direct imprint (LADI) [190]. This method has the advantages of high-resolution (sub-10 nm) and speed (250 ns for embossing time). A single excimer laser pulse (308 nm wavelength and 20 ns pulse duration) passes through a quartz without absorbing the laser energy because quartz has larger bandgap than the photon energy. Then, solid-state silicon will be in liquid phase when absorbing the laser energy. After laser irradiation, silicon embossing with the quartz mold is done quickly. LADI can achieve not only short process time, but also extend the resist materials such as germanium, polysilicon, and other dielectrics [190]. Cui et al. used a nanosecond pulse laser to fabricate sharp and high aspect ratio metal tips [191]. This method can skip the lift-off, cooling, and etching steps, to improve the throughput. Nagato et al. added diamond-like carbon (DLC) thin films onto the quartz substrate as a layer for light-absorption which is directly heated by the laser irradiation [192]. LADI is a promising imprinting method in various aspects: (1) sub-10 nm resolution; (2) no need for etching; cheaper equipment (no need for focusing optics); and (3) applicable to metal substrates (Figure 10c).

Yokoo et al. developed nanoelectrode lithography (NEL) which combines EBL and imprinting (Figure 6f) [137]. NEL uses a patterned nanoelectrode for the electrochemical reaction on the substrate surface. For this, a nanopatterned conductive mold is essential. The mold contacts the substrate and a voltage is applied through the electrode to the substrate. After that, the contact area reacts, and the pattern is transferred from the conductive mold. Finally, the target is etched or developed. In their following research, they demonstrate that NEL can establish multiple patterning on a gallium arsenide substrate over the large area (6 mm × 8 mm). Multiple patterning is possible to fabricate 3D metamaterial structures. Scaffaro et al. suggested that NEL can be used for mold fabrication too [193].

## 4. Conclusions and Outlook

This review introduced and proposed scalable and high-throughput manufacturing methods of metasurfaces towards practical metadevices that can be used to replace existing bulk optics. The most commonly used optical method of photolithography has the ability to produce subwavelength structures. Its capability depends on the photomask used which produces various and complex nanopatterns. However, each pattern requires the corresponding photomask that is costly and time-consuming to produce. The maskless LIL technique can resolve these concerns. It requires a simple optical system with only several optical components. The limited exposure area in Lloyd’s mirror LIL may be overcome by beam splitting LIL that uses multiple beams. Plasmonic-based interference lithography realizes deep-subwavelength resolution beyond the optical diffraction limit that occurs in LIL and photolithography, thanks to SPPs and BPPs. We also presented imprint methods for large area fabrication. Conventional imprinting methods, sorted by curing sources, contact types, and mold types, were investigated and applied to fabricate metamaterials. Finally, the emerging methods including functional resin and resin-free techniques were introduced and reviewed. At the end of each section, we reviewed the most recent developments of different metadevices using each manufacturing method.

Metasurfaces have been proven to exhibit extraordinary optical properties through various research. In addition, they have the potential to dramatically reduce the physical dimensions of optical devices due to their ultra-thin, small, and light weight features. However, the absence of proper manufacturing methods for the mass production of metasurfaces has been one of the primary obstacles preventing realization of this potential in real world applications. Indeed, there is no obvious method to make practical metasurfaces with both large effective areas and efficient manufacturing speeds as yet, despite the significant efforts of numerous experts [48,194]. However, these efforts have always resulted in steady advances in different fields such as physics, chemistry, optics, and material science. As a result, many nanofabrication methods have been developed, and we selected and reviewed the significantly promising methods here. The typical methods such as conventional photolithography, interference lithography, and plasmonic lithography have their own respective limitations which need to be overcome before they can realize the perfect mass production of metasurfaces. However, the synergy between different fields will continue to improve upon the limitations such as PRL, functional resin-based NIL, and LADI (resin-free imprinting). We believe that these advanced technologies will realize both the extraordinary optical properties of metasurfaces and miniaturization of bulk optical devices, further combined by recent deep learning assisted nanophotonics design methodolology [195,196,197,198,199,200,201,202]. These developments will also enable the replacement of bulk optics with metadevices such as wave-modulation metadevices, metalenses, plasmonic color filters, metaholograms, and so on.

## Figures and Tables

**Table 1:** Comparison of the thermal NIL and ultraviolet (UV) NIL.

Comparing Features	Thermal NIL	UV NIL
Material viscosity	High viscosity in low temperature Low viscosity in high temperature	1 to 100 cP (general material)
Pattern size	Hundred nanometers	<10 nm is possible
Resist coating	Spin-on	Spin-on or drop on demand
Processing time	Slow due to heating and cooling	Fast
Filling driven force	Pressure	Pressure and capillary
Type of materials	More material are possible	Material options are limited

**Table 2:** Comparison of the various types of roller-based NIL.

	Plate-to-Plate Type NIL	Roller-Based NIL	
		Roll-to-Plate NIL	Roll-to-Roll NIL
Mask and substrate	Flat molds (rigid or flexible) with rigid substrate	Roller mold (flexible) with rigid substrate	Roller (rigid or flexible) mold with
Printing area	450 mm × 500 mm	1 m × 0.3 m	300 mm(width)
Resolution	Sub-20 nm	40 nm	Sub-30 nm
Throughput	Moderate	High	Very high
Contact mechanism	Surface contact	Line contact	Line contact
Limitation	High pressure, large-area demolding	Conformal contact	Roller mold fabrication, coating method
